# Assessing the Flavor of Various Edible Meats Including Wild Raccoon Meat by the Check-All-That-Apply Method

**DOI:** 10.3390/foods14132191

**Published:** 2025-06-23

**Authors:** Wataru Mizunoya, Nanami Hayashi, Asuka Kataoka, Hinako Nishikawa, Minori Todoroki, Chihiro Kase, Shiro Takeda

**Affiliations:** Department of Animal Science and Biotechnology, School of Veterinary Medicine, Azabu University, Sagamihara 252-5201, Japan

**Keywords:** meat, raccoon, Check-All-That-Apply (CATA), sensory evaluation

## Abstract

Consumers often demonstrate an intrinsic aversion to unfamiliar foods referred to as food neophobia. Wild raccoon (*Procyon lotor*) meat represents such an unfamiliar food, both globally and within Japan. Providing information on the flavor of raccoon meat may help to alleviate consumer apprehension. Here, we employed the Check-All-That-Apply (CATA) sensory evaluation method to delineate its flavor profile. Ground meat samples of beef, pork, chicken, lamb, tuna, frog, venison, and raccoon were prepared using a food processor. The meat colors exhibited marked variation, with raccoon meat characterized by its notably high redness and low lightness. All meat samples were steam-cooked for 15 min prior to evaluation. Sensory assessment was conducted using the CATA method, wherein 17 Japanese panelists selected among 27 descriptors for flavor/taste and texture. The collected data were analyzed through correspondence analysis, revealing that raccoon meat was primarily characterized by the descriptors “having aftertaste,” “rich,” “difficult to swallow,” “lamb/mutton-like,” “gamey,” and “quirky,” indicating a sensory profile closely resembling that of lamb among the evaluated livestock meats. Thus, the CATA method could be effective in characterizing the flavor profile of raccoon meat, highlighting its similarity to lamb and presenting a viable strategy to familiarize consumers with this unconventional protein source.

## 1. Introduction

There has been increasing interest in the role of diet in promoting overall health. The nutritional benefits of game meat are becoming more widely recognized, leading to a growing demand for this alternative protein source [1]. Game meat is distinguished by its optimal chemical composition, low fat content, and a favorable ratio of unsaturated to saturated fatty acids, as well as a high protein content [2,3,4,5,6] and well-balanced amino acid profile [7,8] which contribute to its nutritional value. As a result, game meat holds promise as a viable alternative to conventional livestock meat, depending on consumer preferences and dietary needs. However, despite this rising interest, its consumption remains a marginal fraction of total meat intake globally [9] and in Japan [10], significantly lagging behind that of traditional livestock species.

The raccoon (*Procyon lotor*) is a medium-sized mammal native to North America, classified within the order Carnivora. It was introduced into the wild in Japan in the 1960s [11]. According to a survey conducted by the Ministry of Agriculture, Forestry and Fisheries of Japan, raccoon-related crop damage had increased more than tenfold by 2023 compared with the year 2000 [12]. Raccoons primarily eat vegetable and fruit crops [13,14]; however, as omnivorous predators, they have also been reported to prey on endangered native species, including aquatic amphibians and reptiles [15]. The annual number of raccoons hunted in Japan has surged in recent years, increasing from approximately 10,000 in 2006 to over 50,000 in 2017 [16]. Despite the remarkable rise in the raccoon population, no governmental initiatives have been implemented to promote their utilization as a food resource, in contrast to wild boar and deer, which cause greater agricultural damage and are systematically harvested for meat.

In Japan, raccoon meat is only dealt in limited quantities by wholesalers and restaurants specializing in wild game cuisine. Historically, raccoon meat has been incorporated into traditional recipes in North America for centuries, demonstrating its viability as a consumable protein source [17]. Nevertheless, it remains largely unfamiliar to most consumers. As a potential alternative to conventional livestock, raccoon meat could be considered among the underutilized wild game species. However, there is currently a lack of reliable, objective data characterizing its flavor and taste. Consumers often exhibit an inherent reluctance toward unfamiliar foods, which is known as food neophobia [18]. Providing comprehensive information on the sensory attributes of raccoon meat may help alleviate consumer apprehension. Ultimately, it is anticipated that raccoon meat will gain consumer acceptance and contribute to its widespread utilization.

The sensory attributes of food play a crucial role in shaping consumer preferences [19]. Descriptive Analysis (DA) is a widely utilized, sophisticated, and effective method for characterizing the sensory properties of food [20]. However, DA is a labor-intensive and time-consuming process, requiring extensive effort from panelist selection to final data analysis [21]. To address these limitations, various alternative sensory evaluation methods have been developed, validated, and optimized [22]. Among these, the free permutation task, projection mapping, and Check-All-That-Apply (CATA) methodologies have garnered significant attention within the scientific community [23].

The CATA methodology utilizes a set of descriptive words or phrases that allow panelists to indicate relevant attributes of the product under evaluation. Although relatively novel, the CATA method has been employed in animal science to characterize the sensory properties of bologna sausages [24], Jidori (Japanese local chicken breeds) [25], and various pork products [26], including bacon [27], to identify differences in their characteristics. This approach is widely regarded as a valuable tool for assessing food quality.

In the present study, we aimed to characterize the flavor profile of raccoon meat by comparing it with various other animal meats using the CATA method. The findings of this study are expected to provide valuable insights for consumers regarding the potential application of wild game meat as a food resource.

## 2. Materials and Methods

### 2.1. Materials

The raccoon meat used in this experiment was purchased from the commercial retailer (Itoshima Gibier Laboratory, Itoshima, Japan). The meat was processed in accordance with stringent hygienic quality standards and was approved for human consumption. These raccoons were captured using small box traps in Soeda Town, Fukuoka Prefecture, between May and June 2023. The raccoon meat was delivered to our laboratory as frozen carcasses. After thawing, lean meat was meticulously excised to the greatest extent possible before being refrozen. Beef (New Zealand beef sirloin), pork (Japanese pork loin), lamb (New Zealand lamb short loin), chicken (Japanese young chicken meat), venison (Japanese deer loin), and frog meat (Chinese bullfrog leg) were purchased in either chilled or frozen form from the commercial retailer (Kawashima Food Co., Ltd., Tokyo, Japan). Additionally, red tuna meat was obtained from a local retail shop. All meat samples were stored in a −20 °C freezer and thawed in a 4 °C refrigerator prior to processing into ground meat.

### 2.2. Preparation of Meat

The thawed meat chunks were trimmed, cut into bite-sized pieces, and processed in a food processor for 30 s to obtain ground meat. Since it was impractical to process all the meat simultaneously, the ground meat was vacuum-sealed in small portions and stored frozen. On the day before sensory evaluation, ground meat from eight different animal species was thawed simultaneously in a refrigerator at 4 °C. The thawed meat was weighed to approximately 30 g, mixed with 0.7% (*w*/*w*) sodium chloride, and molded into 90 mL metal cups to ensure uniformity across samples. The meat samples were wrapped in plastic film, placed in a preheated food steamer (STM-1000J, Conair Japan G.K., Tokyo, Japan), and steamed for 15 min. Prior to the sensory evaluation, the core temperature of the meat during cooking was monitored using a small button-type temperature logger (Hyperthermochron, #1922E, KN Laboratories Inc., Ibaraki, Japan). After steaming, the plastic film was removed, and each meat sample was cut into eight pieces using a knife. To prevent drying, the cooked meat pieces were stored in plastic cups with lids and maintained at 60 °C in a thermostatically controlled incubator until just before serving for sensory evaluation.

### 2.3. pH and Meat Color

The pH values of ground meat were determined with a pH meter (testo 206-2, Testo Co., Yokohama, Japan). The raw ground meat color was measured after a 30 min minimum bloom time with a CR-13 Color Reader (Konica Minolta Sensing Inc., Osaka, Japan). The lightness (L*), redness (a*), and yellowness (b*) were measured from three different locations. From these color indices, the angular coordinates of chroma (C*) and hue angle (h*) were calculated using the following formulas: C* = ((a*)^2^ + (b*)^2^)^1/2^ and h* = arctan (b*/a*), in which h* was converted from radians to degrees [26].

### 2.4. Selection of Panelists

The study’s objectives and procedures were thoroughly explained to all participants, and only those who provided informed consent were included. Initially, 31 untrained volunteer participants, comprising Japanese laboratory students and staff members (16 males and 15 females aged between their 20s and 40s), took part in the panelist selection process. Participants underwent a specialized sensory assessment to evaluate their ability to recognize basic tastes. Five standard taste solutions representing saltiness, sweetness, bitterness, sourness, and umami were prepared using food-grade compounds: sodium chloride (Tomita Pharmaceutical Co., Ltd., Naruto, Japan) for saltiness, granulated sugar (Wellneo Sugar Co., Ltd., Tokyo, Japan) for sweetness, anhydrous caffeine (Shiratori Pharmaceutical Co., Ltd., Chiba, Japan) for bitterness, citric acid (Kanto Chemical Co., Inc., Tokyo, Japan) for sourness, and monosodium glutamate (Mitsubishi Corporation Life Sciences Limited, Tokyo, Japan) for umami. These solutions were formulated based on ISO 3972 standards [28], with slight modifications to the concentrations outlined in Table 1. The lowest concentration detected by trained panels in ISO 3972 was adjusted to fall within a range between half-threshold and threshold levels. Participants were presented with 30 mL aqueous solutions of each basic taste—sweetness, saltiness, sourness, bitterness, and umami—and a tasteless control (water) in a blinded test. They were instructed to identify the taste or the water sample. The tests were conducted in the following order: half-threshold, threshold, and twice-threshold concentration. The selection criterion was set at a minimum accuracy rate of 70% in correctly identifying the samples. As a result, 17 participants (5 males and 12 females) met the criteria and were selected as panelists for the sensory evaluation.

### 2.5. CATA Questionnaire for Sensory Evaluation

To develop the descriptors for the CATA used in the questionnaire, samples of pork, beef, and chicken were prepared in advance for descriptor determination in the pilot study. A total of 27 descriptors for flavor/taste and texture were used in this study (Table 2, original descriptors in Japanese are shown in Supplementary Table S1). Panelists answered the CATA questions composed of 27 descriptors and were asked to check all the descriptors that they considered appropriate to describe the cooked meat samples. As the cooked meat samples were very different in color by animal, there was a concern that the samples could be visually distinguished. Therefore, to obstruct visual information, all panelists ate the meat samples while wearing an eye mask. The panelists were required to ensure that they drank at least one sip of water each time the sample was changed. The evaluations were answered via an electronic device using an online form. The order of sample presentation to the subjects was randomized.

### 2.6. Statistics

The comparison of meat color was analyzed using a one-way analysis of variance (ANOVA), followed by Tukey’s test, conducted with EZR (version 1.68, Saitama Medical Center, Jichi Medical University, Saitama, Japan), a graphical user interface for R (The R Foundation for Statistical Computing, Vienna, Austria). A *p*-value of less than 0.05 was considered statistically significant. For the CATA method, differences in the frequency of selected evaluation terms among samples were assessed using Cochran’s Q test. Correspondence analysis for the CATA data was performed within the R environment using the SensoMineR package (version 1.27) [29].

## 3. Results

### 3.1. Core Temperature of Meat Samples During Cooking

**Figure 1:**
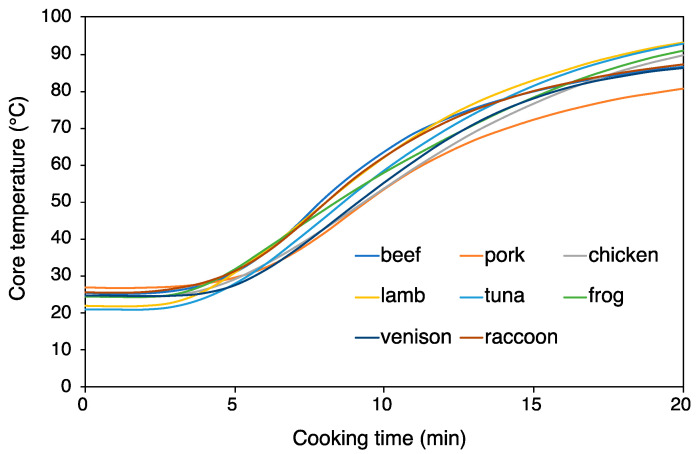
Time-course change of core temperatures during steam cooking of eight different meat samples.

Core temperatures of meat samples during steam cooking were measured to ensure that they were adequately heated for sensory evaluation. Preliminary experiments indicated that maintaining a consistent core temperature across meat blocks from different animal species under uniform heating conditions was challenging due to variations in thickness and size. To address this, various cooking methods were tested, and it was determined that steam cooking using ground meat of standardized weight ensured uniform heating across different species, making it an optimal method for temperature control (Figure 1). Since the core temperature reached at least 72 °C after 15 min of steam cooking, the cooking duration in this experiment was set to 15 min. This cooking condition exceeded the FDA Food Code criteria for wild game animals [30].

### 3.2. pH and Meat Color

**Figure 2:**
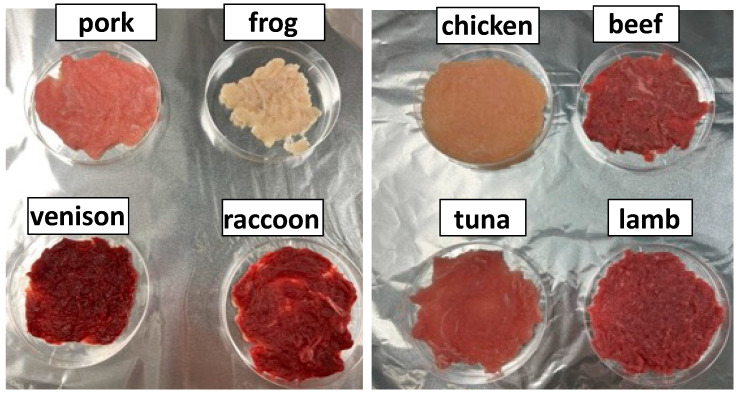
Visual color of the eight different raw ground meat samples.

The pH values of ground raw beef, pork, chicken, lamb, tuna, frog, venison, and raccoon meat were 5.82, 5.80, 5.78, 5.99, 5.71, 6.10, 5.65, and 6.32, respectively. High-quality meat typically exhibits an ultimate pH in the range of 5.4–5.6 [31]. Most of the meat samples analyzed in this study had slightly higher pH values than the standard range, with particularly elevated levels observed in frog and raccoon meat. A photograph of the raw meat samples is presented in Figure 2, while the raw meat color scores are detailed in Table 3. The a* value, an indicator of redness, was similarly high in beef, lamb, venison, and raccoon meat. In contrast, the L* value, which represents lightness, exhibited an inverse trend, being lower in beef and lamb. The L* values for venison and raccoon meat were even lower than those of other species. Although significant differences were observed, the b* value, which indicates yellowness, did not vary markedly among the samples. Chroma (C*) and hue angle (h*), both derived from a* and b* values, also differed among the meat samples. Chroma (C*), which quantifies color saturation, was notably high in beef, lamb, venison, and raccoon meat, though to a lesser extent than a* values, as the b* values exhibited relatively minor differences. The hue angle (h*) varied across samples, indicating differences in overall color tone, which is a combination of redness and yellowness.

### 3.3. Sensory Characterization of the Meat Samples

**Figure 3:**
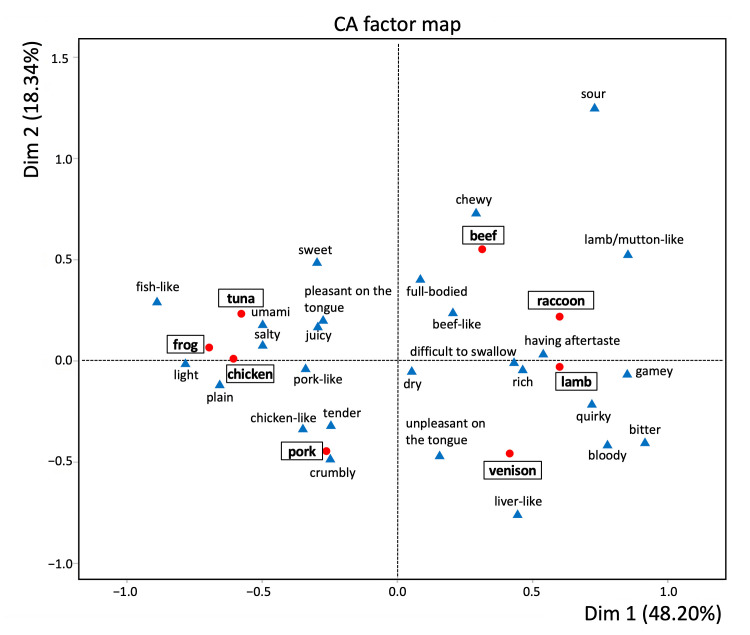
Correspondence analysis (CA) factor map of the sensory descriptors on the Check-All-That-Apply (CATA) questionnaire for the eight different meat samples (beef, pork, chicken, lamb, tuna, frog, venison, and raccoon).

We conducted a sensory evaluation test using the CATA method. The frequency with which each evaluation descriptor was selected by panelists was recorded for each sample (Table 4). Cochran’s Q test identified 17 descriptors with significant differences across samples (*p* < 0.05). Raccoon meat was frequently associated with the descriptors “quirky,” ”gamey,” and “chewy.” A correspondence analysis of descriptor frequencies is presented in Figure 3 as a two-dimensional perceptual map. Samples exhibiting similar sensory characteristics are plotted in close proximity, while descriptors frequently associated with specific samples are also clustered together. Furthermore, descriptors positioned in the same directional plane as a given sample can be interpreted as key attributes defining that sample’s characteristics. The contribution of the primary component was 48.2% and that of the secondary component was 18.3%, meaning that the sum of the primary and secondary components can explain 66.5% of the total data. Raccoon meat was primarily characterized by the descriptors “having aftertaste,” “rich,” “difficult to swallow,” “lamb/mutton-like,” “gamey,” and “quirky,” indicating a sensory profile closely resembling that of lamb among the evaluated livestock meats.

## 4. Discussion

The aim of this study was to evaluate and facilitate the understanding of raccoon meat’s taste, which remains unfamiliar to many, by providing an analogy that would be comprehensible even to those with no prior experience. Correspondence analysis of the CATA method revealed that raccoon meat was perceived similarly to lamb. Raccoon meat exhibited a high selection rate for the descriptors “quirky” and “gamey,” indicating that it possesses a distinctive aroma compared with other animal species. Traditionally, the flavor of unfamiliar meats could only be inferred through the subjective judgment of experienced individuals. However, the application of the CATA method allowed individuals with no prior exposure to raccoon meat to conceptualize its taste. Furthermore, this approach provided statistical and visual insights into how such unfamiliar meats compare to more commonly consumed varieties.

Analysis of the pH of the raw meat showed that raccoon meat had a significantly higher pH than 6. A high ultimate pH (above 6) has been recognized as a characteristic of dark, firm, and dry (DFD) meat [32]. The underlying cause of the elevated pH in raccoon meat remains unclear; however, it is hypothesized that glycogen depletion in skeletal muscle due to pre-slaughter stress inhibits post-mortem lactic acid production, leading to a high ultimate pH. This condition is akin to DFD meat, which is considered an anomaly in livestock. Similar trends have been observed in other game meats, such as wild boar and red deer [2], where a subset of captured animals has been reported to exhibit high ultimate pH values. Two large-scale studies on red deer (*n* = 3500; New Zealand) [33] and reindeer (*n* = 3400; Sweden) [34] found that 11% and 29% of carcasses, respectively, had a meat pH exceeding 5.8, suggesting that such variations may be inevitable in wild animal harvesting. However, previous sensory evaluations have indicated that high-pH meat is often perceived as more tender and juicier than normal [35]. The primary concern regarding meat quality under these conditions is its increased susceptibility to microbial proliferation. Nonetheless, microbial growth can be effectively managed through temperature control and the implementation of stringent hygienic measures during meat processing in modern facilities.

Raccoon meat has an intense reddish color, similar to venison among game meats and beef and lamb among domesticated species. This pronounced red coloration can be attributed to its high myoglobin content. For instance, Mori et al. reported that the distinct coloration of deer sausage was directly linked to the elevated myoglobin concentration in raw deer meat (deer: 0.63%; pork: 0.29%) [36]. Not only deer but game meats in general tend to have higher myoglobin levels, resulting in darker coloration and subsequently higher iron content compared with domestic meats [37,38,39]. Given its myoglobin content, raccoon meat is likely a valuable source of dietary iron, comparable to other wild game, with publicly available databases estimating an iron content of 6.04 mg per 28.3 g (1 oz) of cooked meat [40]. However, the comprehensive nutritional profile of raccoon meat remains largely unexplored, warranting further investigation in future studies.

Current research on the meat quality and flavor profile of raccoon meat appears to be limited. This may be attributed to the fact that raccoon meat is not widely consumed on a global scale. However, the increasing number of hunted raccoons presents a potential opportunity for utilizing their meat as a food source. In the United States, raccoon meat is part of traditional cuisine [17], albeit less commonly consumed than other game meats. Furthermore, studies suggest that hunters are more likely to consume raccoon meat compared with non-hunters [41,42,43].

One of the limitations of this study pertains to the inherent variability in raccoon meat quality. It remains uncertain whether the characteristics of the raccoon meat analyzed in this study are representative of the species as a whole. Due to climatic differences, geographical conditions, and dietary composition, the chemical composition, physical properties, and sensory attributes of game meat can exhibit substantial variability. Moreover, the nutritional value of game meat is influenced by multiple factors, including species, sex, age, body condition, physiological state, hunting season (e.g., fat accumulation in preparation for winter), foraging habitat, ambient temperature, and the specific anatomical part of the carcass [5]. Further research is necessary to elucidate the extent of variation in the meat quality and nutritional composition of raccoon meat.

Another limitation of this study was the small size of the sensory evaluation panel. One reason for this was the limited amount of raccoon meat available. The recommended minimum number of participants for CATA is 60–80 [21,44]. It has also been suggested that when the number of participants falls below 30, a non-discriminatory sample profile may emerge [45]. However, few studies have examined how the degree of difference between samples affects the minimum number of consumers required for stable results in statistical analyses. It is thought that a larger difference between samples may reduce the required number of consumers [46]. Alexi et al. also reported that even a short training period can make CATA results more consistent with those from DA [47]. They further stated that the training of participants can reduce the required number of CATA participants. In our study, a screening taste test was used to select only subjects with a high ability to discriminate flavors. This process can be considered a form of training. Therefore, it is expected that the detection power could be comparable to that obtained using a larger number of untrained participants. However, further research is needed to confirm this assumption.

Parasitic infections represent a primary focus in raccoon research. In such studies, the emphasis is placed not on the consumption of meat but rather on the role of raccoons as intermediate hosts for parasites. For instance, toxoplasmosis is a globally prevalent zoonotic disease caused by the protozoan parasite *Toxoplasma gondii*, which has the capacity to infect warm-blooded vertebrates, including humans. Engel et al. reported that the seroprevalence of *T. gondii* in raccoons has been reported to range from 13% to 84.4% in North America, and approximately 37.4% in Germany [48]. In Japan, Sato et al. reported a seroprevalence of 9.9% in wild raccoons [49]. Thermal inactivation of *T. gondii* cysts in meat is considered effective at 67 °C, a temperature that was greatly exceeded in this study.

## 5. Conclusions

In this study, a sensory evaluation was conducted using the CATA method to assess various types of animal meat, including beef, pork, chicken, lamb, tuna, frog, venison, and raccoon meat, which was considered a novel culinary experience. Correspondence analysis of the CATA data revealed that raccoon meat exhibited visual similarity to lamb. Furthermore, the analysis indicated that for consumers with no prior experience consuming raccoon meat, its taste profile was perceived as comparable to that of lamb. The findings of this study may contribute to increasing consumer awareness regarding the viability of wild game meat, particularly raccoon meat, as an alternative protein resource. By providing empirical data on its sensory characteristics and acceptability, the study may also help reduce food neophobia toward raccoon meat.

## Figures and Tables

**Table 1:** Formulations of five basic taste solutions for screening taste test (%).

Taste	Compound	Half Threshold	Threshold	Twice Threshold
Sweetness	sucrose	0.25	0.5	1.0
Saltiness	sodium chloride	0.1	0.2	0.4
Sourness	citric acid	0.035	0.07	0.14
Bitterness	anhydrous caffeine	0.024	0.048	0.096
Umami	monosodium glutamate	0.015	0.03	0.06

**Table 2:** Sensory descriptors used in the Check-All-That-Apply (CATA) questionnaire.

Type (num.)	Descriptors
Flavor/taste (19)	‘beef-like’, ‘pork-like’, ‘chicken-like’, ‘fish-like’, ‘lamb/mutton-like’, ‘sweet’, ‘bitter’, ‘salty’, ‘sour’, ‘umami’, ‘plain’, ‘bloody’, ‘rich’, ‘quirky’, ‘having aftertaste’, ‘gamey’, ‘liver-like’, ‘full-bodied’, ‘light’
Texture (8)	‘chewy’, ‘juicy’, ‘tender’, ‘dry’, ‘pleasant on the tongue’, ‘unpleasant on the tongue’, ‘crumbly’, ‘difficult to swallow’

**Table 3:** Meat color indices of the eight different raw ground meat samples.

Color Indices	Beef	Pork	Chicken	Lamb	Tuna	Frog	Venison	Raccoon	Significance
Lightness (L*)	33.13 ± 0.32 a	56.17 ± 0.41 b	56.83 ± 0.47 b	34.30 ± 0.30 a	44.00 ± 1.44 d	55.97 ± 0.87 c	21.67 ± 0.84 d	24.07 ± 0.19 d	***
Redness (a*)	21.27 ± 0.74 ab	9.60 ± 0.26 c	1.63 ± 0.15 d	22.73 ± 0.56 abe	12.97 ± 0.46 c	0.83 ± 0.26 d	24.77 ± 1.33 e	22.87 ± 0.97 ae	***
Yellowness (b*)	14.43 ± 0.15 ab	17.07 ± 1.51 a	14.43 ± 0.15 ab	16.70 ± 0.42 a	16.33 ± 0.69 a	11.13 ± 0.62 b	15.10 ± 0.59 a	14.33 ± 0.41 ab	***
Chroma (C*)	8.45 ± 0.10 a	7.30 ± 0.22 b	5.67 ± 0.03 c	8.88 ± 0.09 a	7.65 ± 0.03 b	4.89 ± 0.12 d	8.92 ± 0.21 a	8.62 ± 0.16 a	***
Hue angle (h*)	34.20 ± 0.74 a	60.38 ± 2.02 b	83.54 ± 0.61 c	36.31 ± 0.71 a	51.50 ± 2.18 d	85.64 ± 1.52 c	31.41 ± 0.71 a	32.11 ± 0.51 a	***

*** *p* < 0.001 by one-way ANOVA. Values within a row with different superscript letters are significantly different at *p* < 0.05 by Tukey’s test. Values are means ± SEM for three different locations.

**Table 4:** Contingency table of the eight meat samples for the 27 CATA descriptors.

Descriptor	Beef	Pork	Chicken	Lamb	Tuna	Frog	Venison	Raccoon	Significance
beef-like	4	2	4	3	0	0	1	2	NS
pork-like	4	5	6	1	1	0	1	0	**
chicken-like	1	7	3	2	3	1	1	1	*
fish-like	1	0	2	1	8	16	2	1	***
lamb/mutton-like	8	0	0	8	1	0	3	7	***
sweet	2	0	1	1	3	1	1	0	NS
bitter	1	0	0	3	3	0	3	0	NS
salty	2	6	5	1	6	6	1	4	*
sour	3	0	0	1	0	0	0	0	*
umami	7	6	12	4	10	7	3	1	NS
plain	2	9	5	1	4	10	1	2	***
bloody	1	1	0	2	0	0	4	2	NS
rich	2	2	1	6	3	0	5	3	NS
quirky	4	4	0	12	1	2	14	12	***
light	2	10	10	1	10	15	2	2	***
having aftertaste	8	4	0	14	4	3	10	8	***
gamey	4	1	0	13	0	2	11	12	***
liver-like	0	5	1	3	0	0	6	3	**
full-bodied	4	1	3	3	1	1	1	2	NS
chewy	13	2	2	5	5	5	0	13	***
juicy	3	2	6	4	11	5	5	4	*
tender	3	13	12	8	11	8	15	2	***
dry	11	12	4	7	2	8	8	5	**
pleasant on the tongue	3	1	6	3	6	6	4	4	NS
unpleasant on the tongue	2	7	1	4	1	2	5	2	NS
crumbly	1	7	3	3	1	8	7	1	**
difficult to swallow	2	4	1	4	0	1	1	5	NS

*** *p* < 0.001, ** *p* < 0.01, * *p* < 0.05, NS indicates that there were no significant differences by Cochran’s Q test.

## Data Availability

The original contributions presented in this study are included in the article/Supplementary Materials. Further inquiries can be directed to the corresponding author.

## Supplementary Materials

The following supporting information can be downloaded at: https://www.mdpi.com/article/10.3390/foods14132191/s1, Table S1: Original descriptors in Japanese.
